# The genome sequence of the red-headed cardinal beetle, *Pyrochroa serraticornis *(Scopoli, 1763)

**DOI:** 10.12688/wellcomeopenres.17362.1

**Published:** 2021-11-23

**Authors:** Duncan Sivell, Natural History Museum Genome Acquisition Lab, Darwin Tree of Life Barcoding collective, Wellcome Sanger Institute Tree of Life programme, Wellcome Sanger Institute Scientific Operations: DNA Pipelines collective, Tree of Life Core Informatics collective, Darwin Tree of Life Consortium

**Affiliations:** 1 Department of Life Sciences, Natural History Museum, London, UK

**Keywords:** Pyrochroa serraticornis, red-headed cardinal beetle, genome sequence, chromosomal, Coleoptera

## Abstract

We present a genome assembly from an individual male *Pyrochroa serraticornis* (the red-headed cardinal beetle; Arthropoda; Insecta; Coleoptera; Pyrochroidae). The genome sequence is 249 megabases in span. The majority (97.92%) of the assembly is scaffolded into 10 chromosomal pseudomolecules, with the X and Y sex chromosome assembled.

## Species taxonomy

Eukaryota; Metazoa; Ecdysozoa; Arthropoda; Hexapoda; Insecta; Pterygota; Neoptera; Endopterygota; Coleoptera; Polyphaga; Cucujiformia; Pyrochroidae; Pyrochroa; *Pyrochroa serraticornis* (Scopoli, 1763) (NCBI:txid346838).

## Background

*Pyrochroa serraticornis* (Coleoptera, Pyrochroidae), the red-headed cardinal beetle, is a medium-large (10–18 mm) dorsoventrally flattened beetle with serrate (female) to pectinate (male) antennae, soft elytra and has an abdomen that widens towards its posterior end. The red head, pronotum and elytra contrast against the black underside, legs and antennae, making this a conspicuous beetle in the field. As with other Pyrochroidae this beetle has a tarsal formula of 5-5-4 with penultimate tarsomeres bilobed ( Cooter, 1991; Unwin, 1984).

*Pyrochroa serraticornis* is widespread across Wales and most of England, with a sparser distribution in northern and south-western English counties ( Alexander *et al.*, 2015; Brock, 2019; Buck, 1954). As a result of its general ubiquity this beetle has been illustrated in many of the popular insect guides ( Brock, 2019; Burton, 1968; Chinery, 1993; Lewington, 2019; McGavin, 2005). *P. serraticornis* is common in woodlands, hedgerows and boundary habitats where there is a supply of decaying wood for the larvae. Adults are often found on herbaceous vegetation within these habitats.

The larvae live under the bark of various tree species where they feed on other insects, fungal hyphae and decaying cambium ( Hůrka, 2005). ( Buck, 1954) highlights oak and beech as host trees while Cooter (1991) adds elm ( *Ulmus* spp.) and a rare observation of swarming males also occurred around an elm stump ( Constantine, 1995). Duffy (1946) reported she often found larvae “in rotten oak logs, felled elms, willows, pear trees etc.”.

*Pyrochroa* larvae are distinctive, dorsoventrally flattened with forward pointing jaws and two prongs (urogomphi) extending backwards from a sclerotised plate at the end of the abdomen ( Cooter, 1991; Duffy, 1946). Molfini *et al.* (2021) report that larvae of *Pyrochroa serraticornis* in central Italy are morphologically different to larvae described from Britain, raising the possibility that across Europe there may be more than one species within this taxon. The genome sequence presented here can be used to investigate whether *P. serraticornis*, as we currently recognise it, actually contains more than one species.

## Genome sequence report

The genome was sequenced from one male *P. serraticornis* collected from Wigmore Park, Luton, UK (latitude 51.88378, longitude -0.36861422). A total of 26-fold coverage in Pacific Biosciences single-molecule long reads and 125-fold coverage in 10X Genomics read clouds were generated. Primary assembly contigs were scaffolded with chromosome conformation Hi-C data. Manual assembly curation corrected 44 missing/misjoins and removed 2 haplotypic duplications, reducing the assembly length by 0.15% and the scaffold number by 32.76%, and increasing the scaffold N50 by 65.39%.

**Figure 1.**
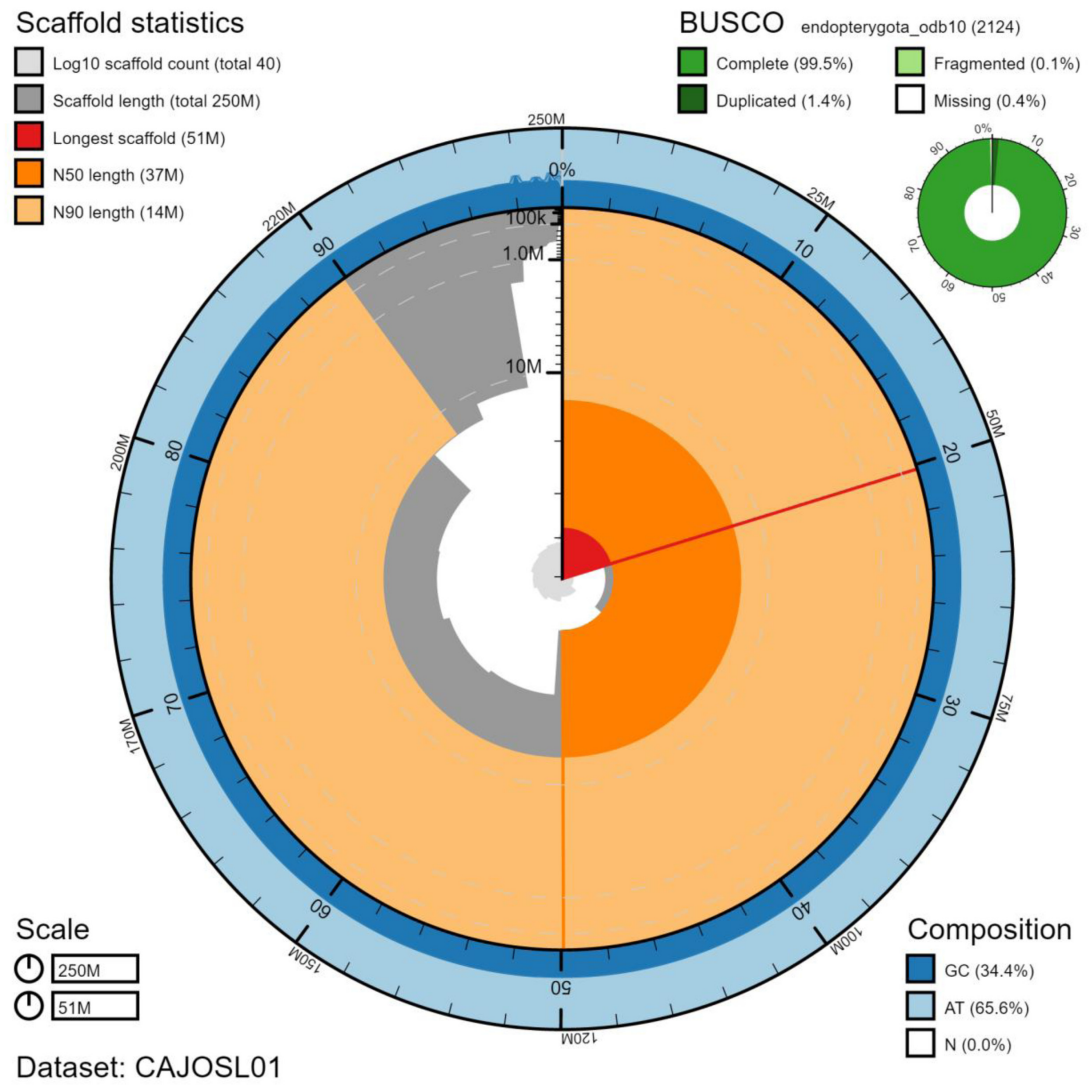
Genome assembly of *Pyrochroa serraticornis*, icPyrSerr1.1: metrics. The BlobToolKit Snailplot shows N50 metrics and BUSCO gene completeness. The main plot is divided into 1,000 size-ordered bins around the circumference with each bin representing 0.1% of the 249,414,617 bp assembly. The distribution of scaffold lengths is shown in dark grey with the plot radius scaled to the longest scaffold present in the assembly (50,529,459 bp, shown in red). Orange and pale-orange arcs show the N50 and N90 scaffold lengths (37,181,734 and 13,582,945 bp), respectively. The pale grey spiral shows the cumulative scaffold count on a log scale with white scale lines showing successive orders of magnitude. The blue and pale-blue area around the outside of the plot shows the distribution of GC, AT and N percentages in the same bins as the inner plot. A summary of complete, fragmented, duplicated and missing BUSCO genes in the endopterygota_odb10 set is shown in the top right. An interactive version of this figure is available at https://blobtoolkit.genomehubs.org/view/icPyrSerr1.1/dataset/CAJOSL01/snail.

The final assembly has a total length of 249 Mb in 39 sequence scaffolds with a scaffold N50 of 37.2 Mb ( Table 1). The majority, 97.92%, of the assembly sequence was assigned to 10 chromosomal-level scaffolds, representing 8 autosomes (numbered by sequence length), and the X and Y sex chromosome ( Figure 1– Figure 4; Table 2). The assembly has a BUSCO v5.1.2 ( Manni *et al.*, 2021) completeness of 99.5% (single 98.1%, duplicated 1.4%) using the endopterygota_odb10 reference set. While not fully phased, the assembly deposited is of one haplotype. Contigs corresponding to the second haplotype have also been deposited.

**Table 1.** Genome data for *Pyrochroa serraticornis*, icPyrSerr1.1.

*Project accession data*	
Assembly identifier	icPyrSerr1.1
Species	*Pyrochroa serraticornis*
Specimen	icPyrSerr1
NCBI taxonomy ID	NCBI:txid346838
BioProject	PRJEB43530
BioSample ID	SAMEA7524259
Isolate information	Male, thorax
*Raw data accessions*	
PacificBiosciences SEQUEL II	ERR6412361
10X Genomics Illumina	ERR6054493-ERR6054496
Hi-C Illumina	ERR6054497
*Genome assembly*	
Assembly accession	GCA_905333025.1
Accession of alternate haplotype	GCA_905333035.1
Span (Mb)	249
Number of contigs	83
Contig N50 length (Mb)	13.0
Number of scaffolds	39
Scaffold N50 length (Mb)	37.2
Longest scaffold (Mb)	50.5
BUSCO * genome score	C:99.5%[S:98.1%,D:1.4%],F:0.1%,M:0.4%,n:2124

*BUSCO scores based on the endopterygota_odb10 BUSCO set using v5.1.2. C= complete [S= single copy, D=duplicated], F=fragmented, M=missing, n=number of orthologues in comparison. A full set of BUSCO scores is available at https://blobtoolkit.genomehubs.org/view/icPyrSerr1.1/dataset/CAJOSL01/busco.

**Figure 2.**
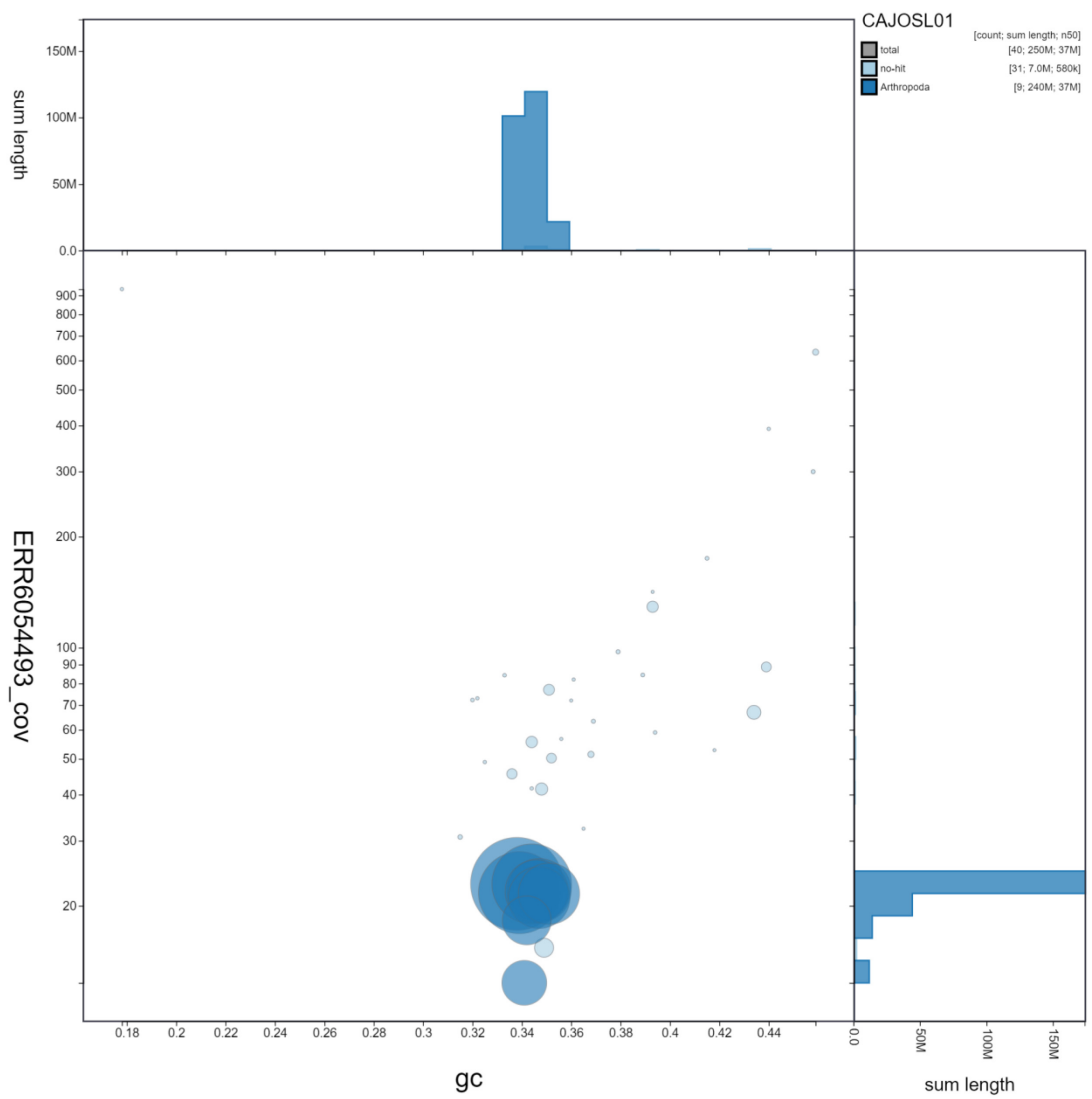
Genome assembly of *Pyrochroa serraticornis*, icPyrSerr1.1: GC coverage. BlobToolKit GC-coverage plot. Scaffolds are coloured by phylum. Circles are sized in proportion to scaffold length Histograms show the distribution of scaffold length sum along each axis. An interactive version of this figure is available at https://blobtoolkit.genomehubs.org/view/icPyrSerr1.1/dataset/CAJOSL01/blob.

**Figure 3.**
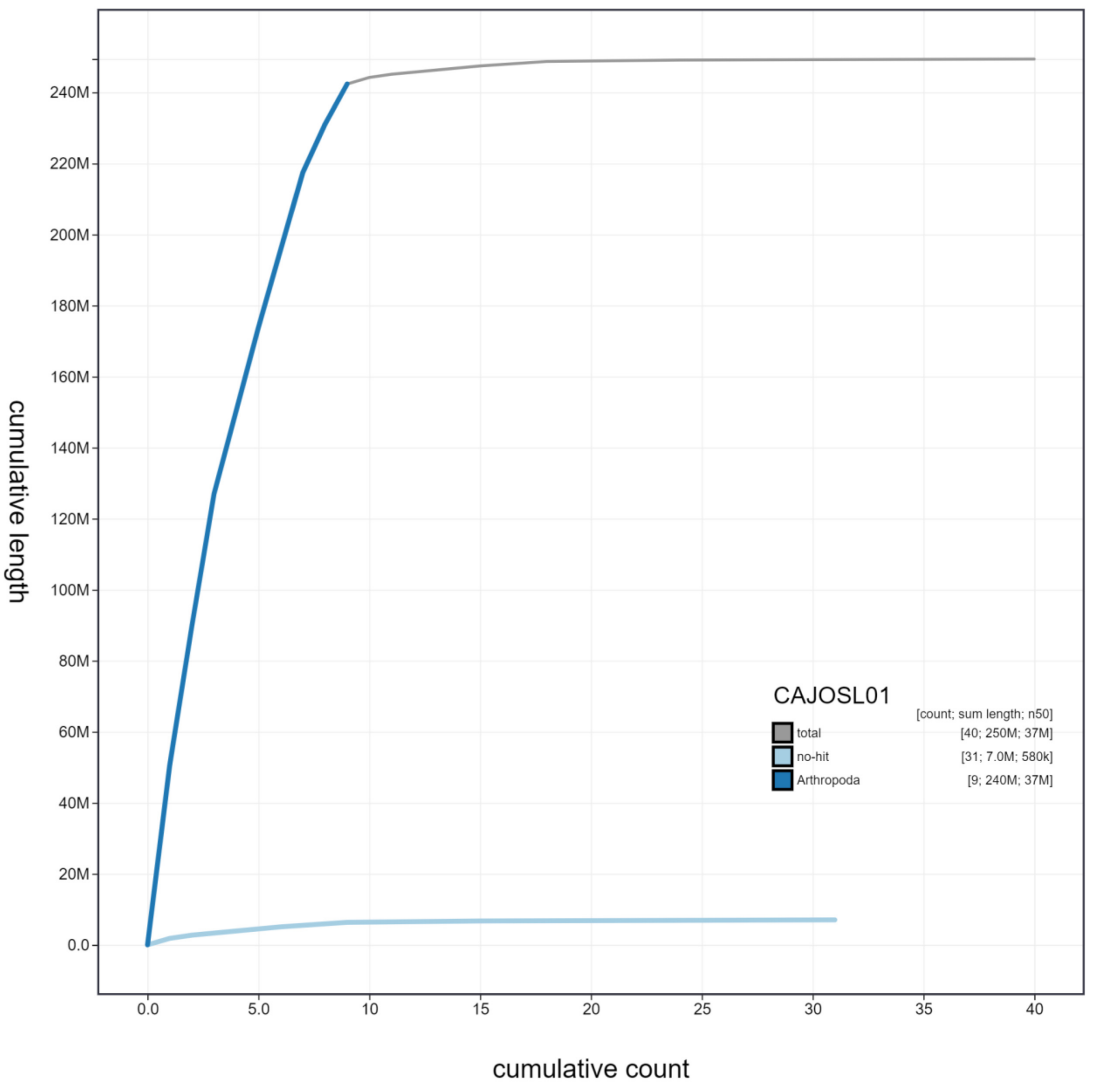
Genome assembly of *Pyrochroa serraticornis*, icPyrSerr1.1: cumulative sequence. BlobToolKit cumulative sequence plot. The grey line shows cumulative length for all scaffolds. Coloured lines show cumulative lengths of scaffolds assigned to each phylum using the buscogenes taxrule. An interactive version of this figure is available at https://blobtoolkit.genomehubs.org/view/icPyrSerr1.1/dataset/CAJOSL01/cumulative.

**Figure 4.**
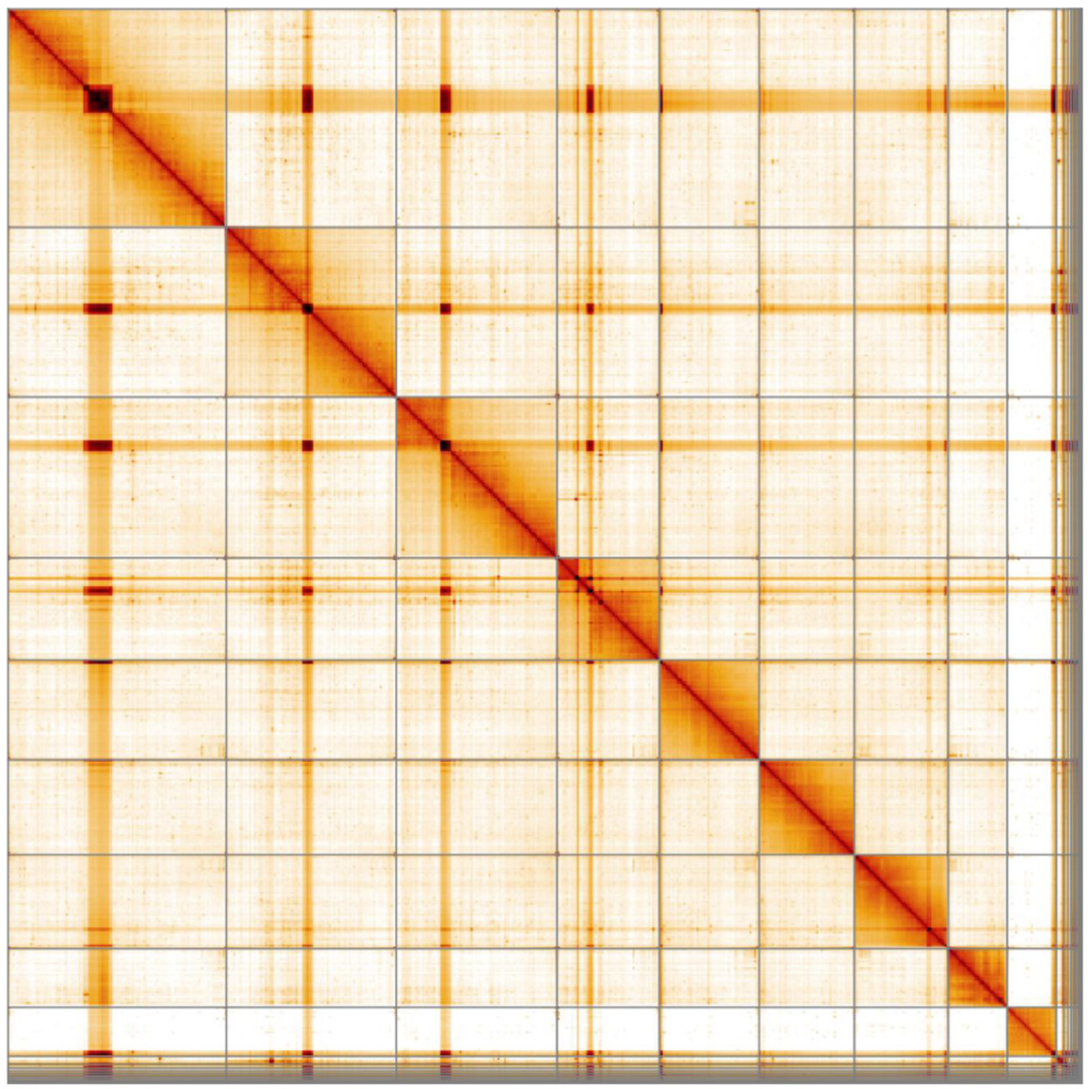
Genome assembly of *Pyrochroa serraticornis*, icPyrSerr1.1: Hi-C contact map. Hi-C contact map of the icPyrSerr1.1 assembly, visualised in HiGlass.

**Table 2.** Chromosomal pseudomolecules in the genome assembly of *Pyrochroa serraticornis*, icPyrSerr1.1.

INSDC accession	Chromosome	Size (Mb)	GC%
HG995152.1	1	50.53	33.8
HG995153.1	2	39.27	33.9
HG995154.1	3	37.18	34.4
HG995155.1	4	23.67	34.7
HG995156.1	5	23.09	34.6
HG995157.1	6	21.99	34.7
HG995158.1	7	21.72	35.1
HG995159.1	8	13.58	34.2
HG995160.1	X	11.35	34.1
HG995161.1	Y	1.83	34.9
HG995162.1	MT	0.02	17.8
-	Unplaced	5.18	37.9

## Methods

A single male *P. serraticornis* was collected from Wigmore Park, Luton, UK (latitude 51.88378, longitude -0.36861422) by Duncan Sivell, Natural History Museum, using a net. The sample was identified by the same individual, and stored at 4°C for 2 hours before preservation on dry ice. Unfortunately, as this specimen was collected during a COVID-19 lockdown, no image was captured prior to preservation.

DNA was extracted at the Tree of Life laboratory, Wellcome Sanger Institute. The icPyrSerr1 sample was weighed and dissected on dry ice with tissue set aside for Hi-C sequencing. Thorax tissue was disrupted using a Nippi Powermasher fitted with a BioMasher pestle. Fragment size analysis of 0.01-0.5 ng of DNA was then performed using an Agilent FemtoPulse. High molecular weight (HMW) DNA was extracted using the Qiagen MagAttract HMW DNA extraction kit. Low molecular weight DNA was removed from a 200-ng aliquot of extracted DNA using 0.8X AMpure XP purification kit prior to 10X Chromium sequencing; a minimum of 50 ng DNA was submitted for 10X sequencing. HMW DNA was sheared into an average fragment size between 12–20 kb in a Megaruptor 3 system with speed setting 30. Sheared DNA was purified by solid-phase reversible immobilisation using AMPure PB beads with a 1.8X ratio of beads to sample to remove the shorter fragments and concentrate the DNA sample. The concentration of the sheared and purified DNA was assessed using a Nanodrop spectrophotometer and Qubit Fluorometer and Qubit dsDNA High Sensitivity Assay kit. Fragment size distribution was evaluated by running the sample on the FemtoPulse system.

### Sequencing

Pacific Biosciences HiFi circular consensus and 10X Genomics read cloud DNA sequencing libraries were constructed according to the manufacturers’ instructions. Sequencing was performed by the Scientific Operations core at the WSI on Pacific Biosciences SEQUEL II and Illumina HiSeq X instruments. Hi-C data were generated from thorax tissue using the Arima Hi-C+ kit and sequenced on an Illumina NovaSeq 6000 instrument.

### Genome assembly

Assembly was carried out with Hifiasm ( Cheng *et al.*, 2021); haplotypic duplication was identified and removed with purge_dups ( Guan *et al.*, 2020). One round of polishing was performed by aligning 10X Genomics read data to the assembly with longranger align, calling variants with freebayes ( Garrison & Marth, 2012). The assembly was then scaffolded with Hi-C data ( Rao *et al.*, 2014) using SALSA2 ( Ghurye *et al.*, 2019). The assembly was checked for contamination and corrected using the gEVAL system ( Chow *et al.*, 2016) as described previously ( Howe *et al.*, 2021). Manual curation ( Howe *et al.*, 2021) was performed using gEVAL, HiGlass ( Kerpedjiev *et al.*, 2018) and Pretext. The mitochondrial genome was assembled using MitoHiFi ( Uliano-Silva *et al.*, 2021). The genome was analysed and BUSCO scores generated within the BlobToolKit environment ( Challis *et al.*, 2020). Table 3 contains a list of all software tool versions used, where appropriate.

**Table 3.** Software tools used.

Software tool	Version	Source
Hifiasm	0.12	Cheng *et al.*, 2021
purge_dups	1.2.3	Guan *et al.*, 2020
SALSA2	2.2	Ghurye *et al.*, 2019
longranger align	2.2.2	https://support.10xgenomics.com/genome-exome/software/pipelines/latest/advanced/other-pipelines
freebayes	1.3.1-17-gaa2ace8	Garrison & Marth, 2012
MitoHiFi	1.0	Uliano-Silva *et al.*, 2021
gEVAL	N/A	Chow *et al.*, 2016
HiGlass	1.11.6	Kerpedjiev *et al.*, 2018
PretextView	0.2.x	https://github.com/wtsi-hpag/PretextView
BlobToolKit	2.6.2	Challis *et al.*, 2020

### Ethics/compliance issues

The materials that have contributed to this genome note have been supplied by a Darwin Tree of Life Partner. The submission of materials by a Darwin Tree of Life Partner is subject to the Darwin Tree of Life Project Sampling Code of Practice. By agreeing with and signing up to the Sampling Code of Practice, the Darwin Tree of Life Partner agrees they will meet the legal and ethical requirements and standards set out within this document in respect of all samples acquired for, and supplied to, the Darwin Tree of Life Project. Each transfer of samples is further undertaken according to a Research Collaboration Agreement or Material Transfer Agreement entered into by the Darwin Tree of Life Partner, Genome Research Limited (operating as the Wellcome Sanger Institute), and in some circumstances other Darwin Tree of Life collaborators.

## Data availability

European Nucleotide Archive: Pyrochroa serraticornis. Accession number PRJEB43530; https://identifiers.org/ena.embl/PRJEB43530.

The genome sequence is released openly for reuse. The *P. serraticornis* genome sequencing initiative is part of the Darwin Tree of Life (DToL) project. All raw sequence data and the assembly have been deposited in INSDC databases. The genome will be annotated and presented through the Ensembl pipeline at the European Bioinformatics Institute. Raw data and assembly accession identifiers are reported in Table 1.

## Author information

Members of the Natural History Museum Genome Acquisition Lab are listed here: https://doi.org/10.5281/zenodo.4790043.

Members of the Darwin Tree of Life Barcoding collective are listed here: https://doi.org/10.5281/zenodo.4893704.

Members of the Wellcome Sanger Institute Tree of Life programme collective are listed here: https://doi.org/10.5281/zenodo.5377053.

Members of Wellcome Sanger Institute Scientific Operations: DNA Pipelines collective are listed here: https://doi.org/10.5281/zenodo.4790456.

Members of the Tree of Life Core Informatics collective are listed here: https://doi.org/10.5281/zenodo.5013542.

Members of the Darwin Tree of Life Consortium are listed here: https://doi.org/10.5281/zenodo.4783559.
