# Supplementary material for: The genome sequence of the red-headed cardinal beetle, Pyrochroa serraticornis (Scopoli, 1763)
Source: Wellcome Open Res. 2021 Nov 23;6:316. [Version 1] doi: 10.12688/wellcomeopenres.17362.1 (PMC11112406; doi:10.12688/wellcomeopenres.17362.1)
Supplement: Peer review history

# Reviewer report: Reviewer response for version 1

**DOI:** 10.21956/wellcomeopenres.19197.r70645

**Published:** 2024-05-11

**Authors:** Manee M Manee

**Affiliations:** 1 King Abdulaziz City for Science and Technology, Riyadh, Saudi Arabia

The study involves constructing a genome assembly of the red-headed cardinal beetle, Pyrochroa serraticornis. The assembled genome spans 249 megabases, with 97.92% scaffolded into 10 chromosomal pseudomolecules, including the X and Y sex chromosomes. A combination of PacBio and Illumina sequencing reads was used. This high-quality genome data is significant for understanding the genetic makeup of this species and potentially identifying more than one species within this taxon.

Are sufficient details of methods and materials provided to allow replication by others?

Yes

Is the rationale for creating the dataset(s) clearly described?

Yes

Are the datasets clearly presented in a useable and accessible format?

Yes

Are the protocols appropriate and is the work technically sound?

Yes

Reviewer Expertise:

bioinformatics, genomics and evolutionary biology

I confirm that I have read this submission and believe that I have an appropriate level of expertise to confirm that it is of an acceptable scientific standard.

# Reviewer report: Reviewer response for version 1

**DOI:** 10.21956/wellcomeopenres.19197.r70644

**Published:** 2024-05-11

**Authors:** Yeon Soo Han

**Affiliations:** 1 Chonnam National University, Cheongju-si, Chungcheongbuk-do, South Korea

The manuscript on "The genome sequence of the red-headed cardinal beetle, *Pyrochroa serraticornis" *is well written and organized with a solid data sets, and thus valuable for the future readers in general and scientific community. It thus appears that it may worth to be acceptable. However, I recommend the authors to rewrite the biological significance of the genome data on *Pyrochroa serraticornis *described in this manuscript.

Are sufficient details of methods and materials provided to allow replication by others?

Yes

Is the rationale for creating the dataset(s) clearly described?

Yes

Are the datasets clearly presented in a useable and accessible format?

Yes

Are the protocols appropriate and is the work technically sound?

Yes

Reviewer Expertise:

Insect molecular biology, Funtional genomics of the beetle insect, Tenebrio molitor, Transciptomics and Genomics

I confirm that I have read this submission and believe that I have an appropriate level of expertise to confirm that it is of an acceptable scientific standard.

# Reviewer report: Reviewer response for version 1

**DOI:** 10.21956/wellcomeopenres.19197.r47201

**Published:** 2021-12-06

**Authors:** Jan Veenstra

**Affiliations:** 1 INCIA, UMR 5287, CNRS, University of Bordeaux, Pessac, France

This short and succinct manuscript describes the genome sequence of *Pyrochroa serraticornis*, commonly known as the red-headed cardinal beetle. The combined used of PacBio and Illumina sequencing on a single male yielded a high quality genome assembly for one haplotype, while contigs for the alternative haplotype have also been assembled. There is really not much more to say, except that one is impressed by the technical advances made over the last two decades that makes it possible to so readily obtain such high quality data. It would have been nice if all the Coleoptera genomes I analyzed two years ago for neuropeptide genes would have been of the same quality.

Are sufficient details of methods and materials provided to allow replication by others?

Yes

Is the rationale for creating the dataset(s) clearly described?

Yes

Are the datasets clearly presented in a useable and accessible format?

Yes

Are the protocols appropriate and is the work technically sound?

Yes

Reviewer Expertise:

NA

I confirm that I have read this submission and believe that I have an appropriate level of expertise to confirm that it is of an acceptable scientific standard.
